# Relative flux trade-offs and optimization of metabolic network functionalities

**DOI:** 10.1016/j.csbj.2022.07.038

**Published:** 2022-07-26

**Authors:** Seirana Hashemi, Zahra Razaghi-Moghadam, Roosa A.E. Laitinen, Zoran Nikoloski

**Affiliations:** a Systems Biology and Mathematical Modeling, Max Planck Institute of Molecular Plant Physiology, Potsdam, Germany; b Faculty of Biological and Environmental Sciences, University of Helsinki, Helsinki, Finland; c Bioinformatics, Institute of Biochemistry and Biology, University of Potsdam, Potsdam, Germany

**Keywords:** Trade-offs, Metabolic networks, Fluxes, Overexpression targets, Growth

## Abstract

Trade-offs between traits are present across different levels of biological systems and ultimately reflect constraints imposed by physicochemical laws and the structure of underlying biochemical networks. Yet, mechanistic explanation of how trade-offs between molecular traits arise and how they relate to optimization of fitness-related traits remains elusive. Here, we introduce the concept of relative flux trade-offs and propose a constraint-based approach, termed FluTOr, to identify metabolic reactions whose fluxes are in relative trade-off with respect to an optimized fitness-related cellular task, like growth. We then employed FluTOr to identify relative flux trade-offs in the genome-scale metabolic networks of *Escherichia coli, Saccharomyces cerevisiae*, and *Arabidopsis thaliana*. For the metabolic models of *E. coli* and *S. cerevisiae* we showed that: (i) the identified relative flux trade-offs depend on the carbon source used and that (ii) reactions that participated in relative trade-offs in both species were implicated in cofactor biosynthesis. In contrast to the two microorganisms, the relative flux trade-offs for the metabolic model of *A. thaliana* did not depend on the available nitrogen sources, reflecting the differences in the underlying metabolic network as well as the considered environments. Lastly, the established connection between relative flux trade-offs allowed us to identify overexpression targets that can be used to optimize fitness-related traits. Altogether, our computational approach and findings demonstrate how relative flux trade-offs can shape optimization of metabolic tasks, important in biotechnological applications.

## Graphical abstract

**Figure:**
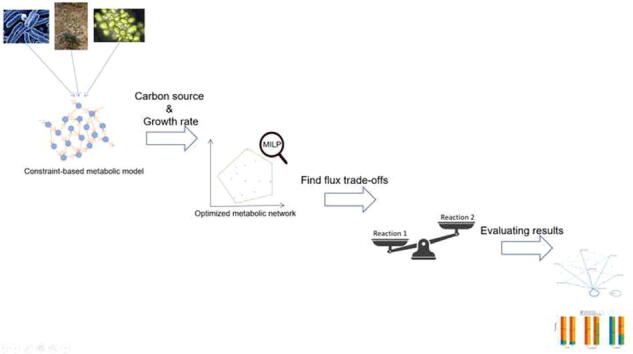

## 1 Introduction

Trade-offs between traits arise in situations when one of the traits can only be increased at the cost of decreasing at least one of the others [1]. Trade-offs are ubiquitous in nature and can be analyzed at and across different levels of biological organization, from the molecular and physiological level to the level of an entire organism and their interactions in eco-systems. For instance, at the molecular level, trade-offs have been reported between expression levels of genes [1], metabolite levels [2], and, recently, reaction fluxes [3]. Examples of trade-offs for focal traits at the level of an entire organism include those between growth rate and yield [4] or growth rate and adaptability [5] in microbes as well as growth and defense in plants [6]. While there is mounting evidence that trade-offs constrain the phenotypes attainable through evolution [7], there is little understanding of how they arise due to the underlying molecular networks that shape different focal traits.

Classically, trade-offs have been explained by the resource acquisition-allocation model, also called Y-model [8], [9]. According to the Y-model two traits, $X_{1}$ and $X_{2}$, in trade-offs are shaped by a common resource, Y, that constrains their values in different environments. More specifically, the Y-model states that $X_{1}+X_{2}=Y$, and as a result $cov\left(X_{1},X_{2}\right)=\frac{1}{2}(var\left(Y\right)-\left(var\left(X_{1}\right)+var\left(X_{2}\right)\right)$. Intuitively, two traits are said to be in trade-off if there are negatively correlated. In the context of the Y-model, a fixed, invariant value (var(Y)=0) for the common resource over different environments implies that its partial allocation to one trait limits the allocation to the other. This then indeed yields a negative correlation between the two traits, and such trade-offs were recently denoted as *absolute* [3]. In addition, in the scenario when the common resource is invariant, the manifestation of absolute trade-offs necessitates that the involved traits exhibit phenotypic plasticity, i.e. their values must vary with the environment, so that the trade-off can be observed [10]. However, using the Y-model, it has also been shown that identifying trade-offs based on covariation can be misleading, since the sign of the correlation between traits is not sufficient to indicate their involvement in a trade-off. For instance, positive correlation is expected whenever the variance of the common resource Y exceeds the sum of variances of the two traits over a set of environments. I Thus, in the case when the common resource in the Y- model exhibits phenotypic plasticity (i.e. var(Y)≠0), the traits in trade-off can also show positive correlation [11], [12]. We will refer to such trade-off situations in which the common resource may vary as *relative* trade-offs.

To overcome the challenge of determining traits in trade-off without relying on covariation, we have recently shown that systematic identification of absolute trade-offs can be achieved by determining a weighted sum of metabolic traits (i.e. fluxes), with non-negative coefficients, that is invariant with the environment. This can be seen as an extension of the Y-model to n traits, whereby $Y=\alpha_{1}X_{1}+{\alpha_{2}X}_{2}+\cdots +{\alpha_{n}X}_{n}$, with all $\alpha_{i}>0$. From this formulation, it is clear that in case when Y is fixed (i.e. $var\left(Y\right)=0$), due to $\alpha_{i}>0$, increase of any trait is accompanied by a decrease of at least one other trait. It is also worth pointing out that, in contrast to the classical Y-model with two traits, here two traits can show positive correlation and still be in trade-off, as described, even when Y is fixed. The reasoning of the Y-model with more than two traits was used as the basis for a constraint-based modeling approach termed FluTO to identify and enumerate absolute flux trade-offs in a given metabolic network under boundary conditions, specifying the environment [3]. By applying flux variability analysis, FluTO first determines reactions with invariant fluxes with the considered boundary conditions. FluTO then identifies weighted sum of fluxes that amounts to an invariant flux, corresponding to the Y resource in the Y-model. FluTO was used with large-scale metabolic networks of *Escherichia coli* and *Saccharomyces cerevisiae* to show that the absolute flux trade-offs are specific to carbon sources and that absolute flux trade-offs in *Arabidopsis thaliana* are dependent on the condition-specific biomass reaction used. However, due to the formulation of FluTO, these results: (i) do not hold in the scenario when the weighted sum of traits is environment-dependent, since the weighted sum equals an invariant flux, and (ii) do not provide insights on how the identified absolute flux trade-offs are related to the optimized fitness-related trait, like growth, since the trade-off was not determined at fixed flux through the biomass reaction.

Here, we formulate a constraint-based approach, termed FluTOr, that allowed us to identify and enumerate relative flux trade-offs with respect to growth simulated by a given metabolic network. More specifically, we are looking for relations of the form $v_{\mathrm{bio}}=\alpha_{1}v_{1}+\alpha_{2}v_{2}+\cdots +\alpha_{n}v_{n}$, with $\alpha_{i}>0$ for at least two fluxes different than the flux of the biomass reaction. The general formulation of FluTOr allows to also enumerate relative trade-offs with respect to other fluxes (e.g. production of a metabolite of interest). This provides larger versatility of FluTOr in comparison to the FluTO used to identify absolute flux trade-offs.

Further, we note that if a relative flux trade-off expresses growth as a weighted sum of reaction fluxes, as stated above, then overexpression of the reactions in trade-off provides a direct way to further increase growth. In other words, if $v_{\mathrm{bio}}$ is not at its optimum, then an increase of any flux $v_{i}$, with $\alpha_{i}>0$, does not have to be compensated by decrease in other fluxes and can lead to increase in $v_{\mathrm{bio}}$. This holds in the cases when the reactions with $\alpha_{i}>0$, implicated in the relative trade-off, show variable flux at higher growth. In contrast to this idea, the existing approaches to enumerate overexpression reaction candidates either rely on time intensive identification of covariation of candidate fluxes with growth, as applied in flux scanning based on enforced objective flux (FSEOF) [13] and its variants [14], or require a reference flux distribution, like in OptReg [15] and GeneReg [16]. In contrast to these approaches, relative flux trade-offs with respect to growth provide new means to identify overexpression targets based on the relation between relative flux trade-offs and growth.

By using FluTOr we then examine the extent to which the identified relative flux trade-offs differ from other seminal concepts in metabolic modeling, namely directional and partial coupling. In addition, we apply FluTOr to large-scale metabolic networks of *Escherichia coli*, *Saccharomyces cerevisiae*, and *Arabidopsis thaliana* to determine relative flux trade-offs with respect to growth and investigate the dependence of the findings on the simulated conditions. As a result, we show that FluTOr provides versatile means to enumerate trade-offs and use them in the identification of overexpression targets to optimize growth. Due to the constraint-based modelling formulation, FluTOr can be readily applied to study and compare trade-offs with respect to other cellular tasks, optimized by evolution, that can be modelled in the context of metabolic networks.

## 2 Methods

### 2.1 Metabolic network models

We apply FluTOr to constraint-based metabolic models of three model organisms, namely the bacterium *E. coli*, the unicellular eukaryote *S. cerevisiae* and the model plant *A. thaliana*. More specifically, we analysed the genome-scale metabolic model iJO1366 of *E. coli* str. K-12 substrain MG1655. This network consists of 1805 metabolites and 2583 reactions [16]. The model has the “core” and “wild-type” biomass reactions. In the simulations of this model we used the “wild-type” biomass reaction [17]. The simulations in yeast relied on the genome-scale metabolic network, yeastGEM v8.3.3, of *S. cerevisiae* with 2691 metabolites and 3963 reactions [18], using the biomass reaction to simulate growth. We used the AraCore model as a representative metabolic network of *A. thaliana*, with 407 metabolites and 549 reactions [19]. The model has three different active biomass reactions: carbon-limiting, nitrogen-limiting, and light-limiting biomass. In the simulations with *A. thaliana* we used one of the three biomass reactions, as detailed in the results.

In all models, we consider different environments, as described in the Results section, by activating a particular carbon and/or nitrogen nutrient. We also considered different lower boundary to modelled growth of 90%, 95%, and 99% of that obtained by flux balance analysis (FBA) [20].

### 2.2 Flux coupling analysis

Two unblocked reactions $r_{i}$ and $\;r_{j}$ are considered coupled if they are directionally, partially, or fully coupled. For completeness, we provide the definitions of the three coupling types: (i) if for all $v\in F,v_{i}\neq 0$ implies $v_{j}\neq 0$ then $r_{i}$ is directionally coupled to $r_{j}$, (ii) if for all $v\in F,v_{i}\neq 0$ implies $v_{j}\neq 0$ and vice versa, then $r_{i}$ and $r_{j}$ are partially coupled; (iii) if $r_{i}$ and $r_{j}$ are partially coupled, and additionally there exists a constant c≠0 such that for all $v\in F,v_{i}\neq 0$ implies $v_{i}/v_{j}=c$, then $r_{i}$ and $r_{j}$ are fully coupled. In any other case, reactions are uncoupled.

### 2.3 Enrichment analysis

Fisher’s exact test is a statistical significant test used to determine if there are nonrandom association between categorical variables. Here, proteins are first divided into two categories based on whether they catalyze only one reaction or more than one reactions. Further, proteins are divided into three categories based on their involvement in trade-offs, namely: proteins for which all reactions are in trade-off, all reactions are not in trade-off, and some reactions are in trade-off. Then the p-value is calculated based on the resulting 2x3 contingency table. More specifically, p=a+bac+dce+fena+c+e, where a,b,c,d,e and f are the number of observations in each cell, n is the total sample size. This test allows us to test the hypothesis that trade-offs are underpinned by promiscuous proteins, catalyzing multiple reactions.

### 2.4 Implementation

FluTOr is implemented in MATLAB and is fully available at https://github.com/seirana/FluTOr.

## 3 Results and discussion

### 3.1 Formulation of FluTOr – A constraint-based approach to identify and enumerate relative flux trade-offs

**Fig. 1:**
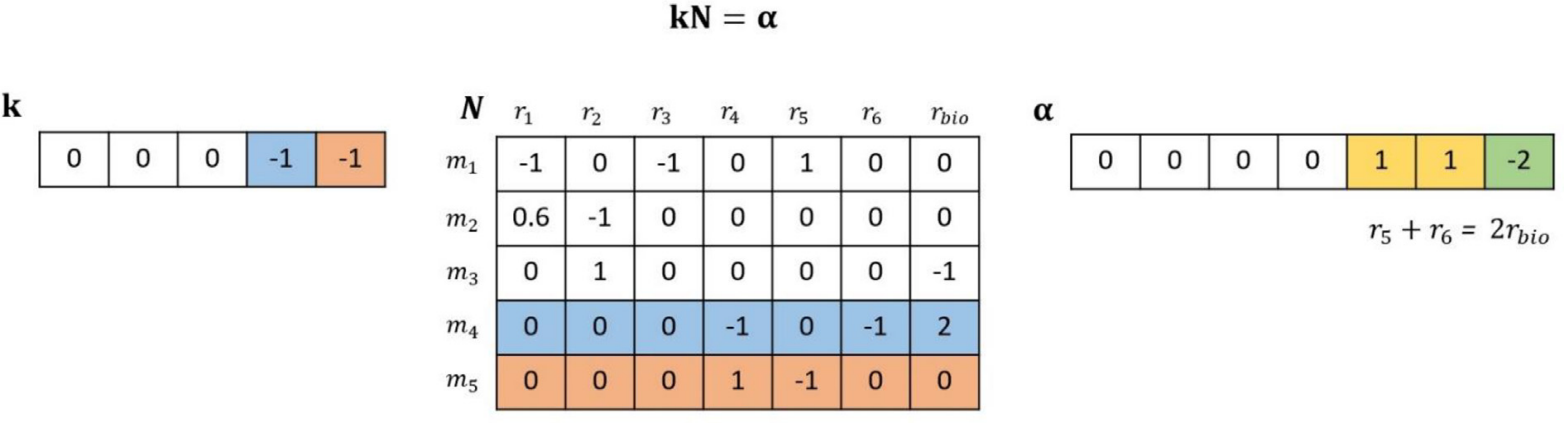
**Illustration of FluTOr**. The approach determines a weighted sum of non-negative fluxes, $\sum_{i=1}^{6}\alpha_{i}v_{i}=v_{\mathrm{bio}}$, with $\alpha_{i}\geq 0$, 1≤i≤6. Here the model has five metabolites and seven reactions, of which one is considered a reaction of interest (in our implementation $r_{\mathrm{bio}})$. From the resulting vector α, it is follows that reactions $r_{5}$ and $r_{6}$ are in a relative trade-off with respect to $r_{\mathrm{bio}}$.

Here, we aim to identify relative trade-offs between reaction fluxes with respect to growth. More specifically, given a metabolic network with n reactions, we seek to determine a weighted sum of non-negative fluxes, $\sum_{i=1}^{n-1}\alpha_{i}v_{i}=v_{\mathrm{bio}}$, with $\alpha_{i}\geq 0$, 1≤i≤n-1. The approach that we describe in this section can in principle be used to specify relative trade-offs with respect to any specified reaction or a subset of reactions. We represent the network by a stoichiometric matrix, N, with m metabolites and n reactions and assume that it operates at a steady-state, whereby Nv=0. The identification of relative trade-offs proceeds in three steps (Fig. 1).

**Fig. 2:**
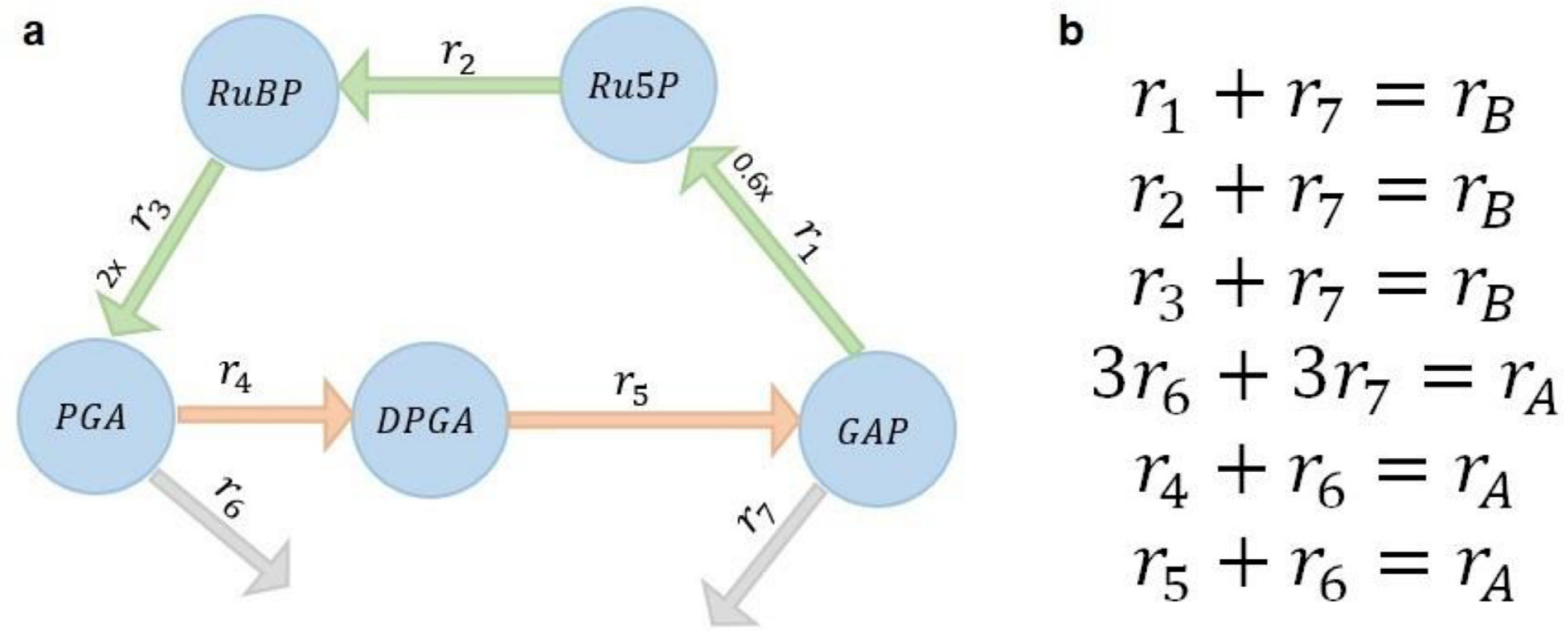
**Illustration of a metabolic network and relative trade-offs.** (a) The metabolic network of the Calvin-Benson cycle is composed of five metabolites (blue nodes), glyceraldehyde 3-phosphate (GAP)*,* ribulose 5-phosphate (Ru5P)*,* ribulose 1,5-bisphosphare (RuBP), diphosphoglycerate (DPGA), and phosphoglycerate (PGA), and seven reactions, denoted by $r_{1}$ to $r_{7}$. The three reactions $r_{1}$, $r_{2}$, and $r_{3}$, represented by green arrows, are fully coupled and are merged in the reaction denoted by $r_{A}$; similarly, the two reactions $r_{4}$ and $r_{5}$ are fully coupled and merged in the reaction $r_{B}$ . We assume that the reactions are irreversible, i.e., the lower bound of are reactions is zero. The upper bound of reactions $r_{1}$ to $r_{7}$ are 600,1000,1000,1000, 600, 200 and 166.7 mmol/gDW/h, respectively. (b) There are six relative trade-offs in the Calvin-Benson cycle that can be identified by solving three MILPs after identifying and merging the fully coupled reactions. (For interpretation of the references to colour in this figure legend, the reader is referred to the web version of this article.)

First, we rely on flux variability analysis (FVA) [21], [22] to categorize the reactions based on the variability in the set of feasible steady-state flux distribution, $F=\left\{v\right|Nv=0,\;0\leq v\leq v_{max}\}$. A reaction is considered blocked if it does not carry flux in any flux distribution in F. A reaction is considered reversible over the flux distributions in F if it takes flux values with different signs. As a result, an irreversible reaction can carry only non-negative fluxes. For instance, a simplified model of the Calvin-Benson cycle—the key subsystem underlying photosynthesis [23]—includes five metabolites and seven irreversible reactions, of which reactions $r_{6}$ and $r_{7}$ are exchange reactions, while the remaining are internal reactions (Fig. 2a). Upon performing FVA, we remove the blocked reactions and dead-end metabolites, and split all identified reversible reactions into two irreversible reactions.

Second, we rely on simplifying the metabolic network by identifying and merging fully coupled reactions based on flux coupling analysis (FCA) [24]. The reaction couplings can be identified by applying quantitative flux coupling analysis (QFCA) [25] and flux coupling analysis (FCA), as implemented in F2C2 [26], both based on solving linear programming problems. Two unblocked reactions $r_{i}$ and $\;r_{j}$ are considered coupled if they are directionally, partially, or fully coupled (see Methods); otherwise, they are uncoupled. The groups of fully coupled reactions, referred to as reaction coupling sets, can be merged into single reactions. For instance, for the network in Fig. 2a, the three reactions $r_{1}$, $r_{2}$, and $r_{3}$ are fully coupled and so are the reactions $r_{4}$ and $r_{5}$; these can be merged in reactions $r_{A}$ and $r_{B}$, respectively. The two export reactions named $r_{6}$ and $r_{7}$ are uncoupled to any other reactions. The merging of fully coupled reactions speeds up the identification of the relative flux trade-offs. For instance, if $\alpha_{i}v_{i}+\alpha_{j}v_{j}=v_{k}$ represents a relative trade-off, with respect to $v_{k}$ and the reaction $r_{i}$ if fully coupled to reaction $r_{l}$, i.e. $v_{i}/v_{l}=c>0$, then ${c\alpha }_{l}v_{l}+\alpha_{j}v_{j}=v_{k}$ also represents a relative trade-off. For instance, a relative trade-off between reactions $r_{1}$ and $r_{7}$ with respect to $r_{B}$ leads to a relative trade-off between reactions $r_{2}$ and $r_{7}$ as well as $r_{3}$ and $r_{7}$ with respect to $r_{B}$ (see Fig. 2b).

As a third step, provided a metabolic network, we formulate a constraint-based approach, FluTOr, to identify relative flux trade-offs with respect to the flux through a specified reaction $r_{j}$ (here, $r_{\mathrm{bio}}$). Let the coefficients of the linear combination of fluxes, α, over F be determined by a vector k, such that kN=α. From the formulation, it follows that the coefficient in the linear combination of fluxes, specified by α, corresponds to a weighted combination of the rows of the stoichiometric matrix, given by kN. To determine α, FluTOr minimizes the number of non-zero entries of kN, such that the flux of reaction $r_{j}$ amounts to the weighted sum of at least two irreversible variable reactions different from $r_{j}$ with positive coefficients. This is captured mathematically by the conditions that $\alpha_{j}<0$, $\sum_{i\neq j}\alpha_{i}\geq 0$, and there are at least three reactions with non-zero values for $\alpha_{i}$, given by $\left|supp\left(\alpha \right)\right|\geq 3$. Since minimizing the support is an NP-hard problem [27], [28], we approximate it by minimizing the first norm of kN, i.e. ${\left\|kN\right\|}_{1}$, resulting in the following convex optimization problem:

$$\min\sum {\left\|kN\right\|}_{1}$$

s.t. Nv=0,

$$kN=\alpha ,\alpha_{j}<0,\;\sum_{i\neq j}\alpha_{i}\geq 0,\left|supp\left(\alpha \right)\right|\geq 3.$$

$$v_{max}\leq v\leq v_{max},$$

Since we aim to enumerate all trade-offs, the formulation above can be readily modified to exclude all previously found solutions (i.e., trade-offs); this is achieved by using integer cuts [29]. The objective function uses the absolute value function, which can be cast as a linear function [30], resulting in a mixed-integer LP (MILP) formulation of FluTOr (see **Supplementary Note**). In the rest of the manuscript, we present the findings from our analyses of relative flux trade-offs with $r_{j}$ corresponding to the biomass reaction, $r_{\mathrm{bio}}$.

### 3.2 Relative flux trade-offs in *E. Coli* and *S. Cerevisiae* are specific to carbon sources

Next, we applied FluTOr to investigate relative flux trade-offs with respect to growth simulated from curated and extensively used large-scale models of *E. coli* and *S. cerevisiae*. Since growth of these microorganisms depends on the carbon source provided, we wanted to examine the extent to which relative flux trade-offs may differ between these environments. In the case of *E. coli* we identified and enumerated the relative trade-offs with respect to growth under 21 carbon sources with ammonium as the sole nitrogen source. For each carbon source we considered three scenarios, corresponding to lower bounds to growth set to 90%, 95%, and 99% of the respective optimum, determined by flux balance analysis (FBA) [20]. While the number of reactions in the identified relative trade-offs varied between 20 and 118 over the carbon source, we found that the maximum number of reactions participating in relative trade-offs was twelve, irrespective of the carbon source. Increasing the lower bound on growth led to an increase in the number of reactions in relative trade-off for 52% of the carbon sources. In contrast, for 20% of the carbon sources this led to a decrease in the number of reactions in relative trade-offs. Last, for 10% of carbon sources, increasing the lower bound on growth resulted in no change in the reactions in trade-off. For the remaining carbon sources, there was no obvious pattern of response. These findings can be explained by blocking of one direction of reversible reactions as the lower bound on growth is increased (Table S1).

The metabolic network of *E. coli* includes 2583 reactions, of which sixteen (<1%, all from the cofactor and prosthetic group biosynthesis subsystem) appeared in all relative trade-offs. This is in line with the essential role of cofactors for cell growth. In addition, almost two thirds (i.e. 1659) of reactions did not appear in any relative trade-offs, while the rest (31%) were blocked (Table S2).

**Fig. 3:**
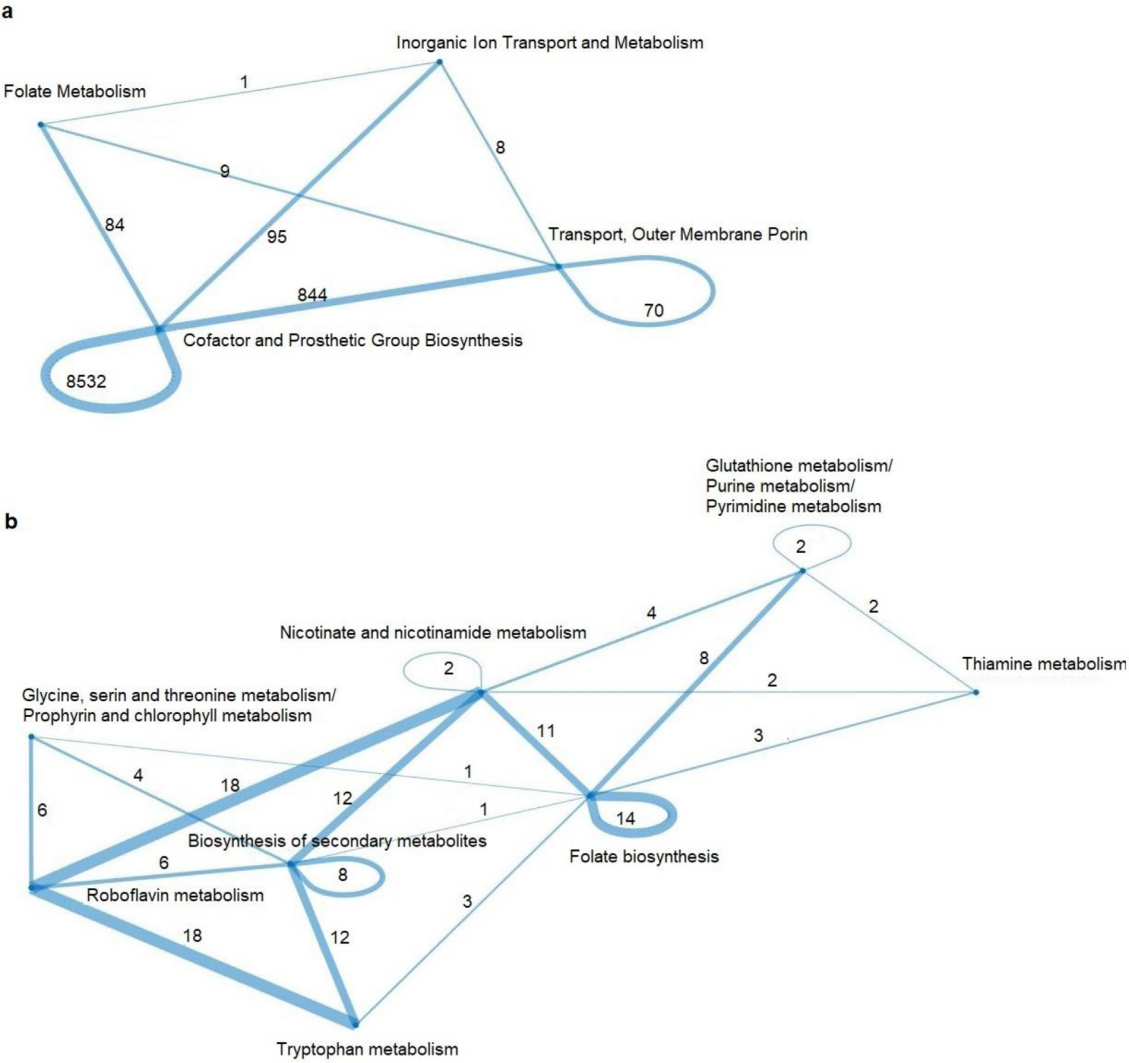
**Subsystems connectivity based on pair of reactions in a relative trade-off under different carbon sources.** Nodes denote metabolic the subsystems and weight of the edges indicate the number of pairs of reactions in relative trade-offs belong to the two connected subsystems in the metabolic models of (a) *E. coli* and (b) *S. cerevisiae*.

We will consider a metabolic subsystem to be *always in trade-off* if at least one of its reactions participates in all of the identified relative trade-offs; it is considered *sometimes in trade-off* if at least one of its reactions participates in some, but not all relative trade-offs. Lastly, a metabolic subsystem is considered *never in trade-off* if none of its reactions participates in any of the identified relative trade-offs. We found three subsystems, namely folate metabolism, inorganic ion transport and metabolism as well as outer membrane porin and transport subsystems to be sometimes in trade-off, while the rest (i.e. 90%) of the subsystems were never in relative trade-offs (Table S3, Fig. 3a). This finding indicates that the alternative (sub)optimal space with respect to growth in *E. coli* show little flexibility, and is targeted at few reactions which may be used to increase growth.

Looking at the proteins associated with the reactions in trade-offs, we categorized them into those that catalyzed only reactions in trade-offs and those that, in addition, catalyze some reactions that did not participate in any trade-offs. The number of proteins that were associated with reactions that did not appear in relative trade-offs decreased from 73% to 65% by increasing the lower bound imposed on growth from 90% to 99%. There were few proteins that were associated simultaneously with reactions that take part in some or were not involved in any trade-offs, and these proteins were dependent on the available carbon source. For instance, with D-manose as a carbon source, nine proteins catalyze only reactions that appeared in trade-offs and two proteins that, in addition, catalyze reactions that did not participate in any trade-offs. On the other hand, with L-arabinose as a carbon source, 12 proteins catalyze only reactions that were in trade-offs, while 61 (84%) were associated to some reactions that did not participate in any trade-offs (Table S4). To determine the statistical significance of these findings, we divided the proteins into those that catalyze only one or multiple reactions; in addition, we also divided the proteins into those that catalyze only reactions in trade-off, only reactions not in trade-off, or both types of reactions. Using the Fisher exact test, we found, in line with expectations, that trade-offs are underpinned by promiscuous proteins that catalyze multiple reactions, indicated by the enrichment analysis (Table S5, **Subsection 2.2**).

Next, we asked if the identified relative trade-offs are species-specific. To answer this question we repeated the analysis with the metabolic model of *S. cerevisiae* with 3963 reactions. We found that in *S. cerevisiae*, the identified trade-offs for the thirteen different carbon sources resulted in, on average, 47 (1.7% of non-blocked) reactions in relative trade-offs. Further, the minimum and maximum of reactions in relative trade-offs over the thirteen carbon sources were 11 and 89, respectively. Almost 30% of reactions were blocked for all carbon sources. Interestingly, we found that there was no reaction in trade-off for all simulated carbon sources, but 3% (117 of 3963) of reactions were in trade-off in at least one carbon source (Table S6).

The reactions that took part in trade-offs participated in 13% (11 of 87) of subsystems (Table S7). Interestingly, none of the metabolic subsystems was found to be in trade-offs across all carbon sources, i.e. the involvement of metabolic subsystems in trade-offs depended on the provided carbon source. Like in *E. coli*, the metabolic subsystems that harbored reactions in flux trade-offs included folate metabolism as well as biosynthesis of cofactors and prosthetic groups, including riboflavin and thiamine metabolism (Table S8). However, reactions in relative trade-offs in *S. cerevisiae* were also found in metabolism of amino acids, e.g. glycine, serine, and threonine metabolism as well as tryptophan metabolism. As a result, reactions involved in biosynthesis of secondary metabolites were also included in relative trade-offs. The sharing of reactions between the eleven metabolic subsystems that harbor trade-offs is depicted in Fig. 3b.

In addition, we found that about 7% of proteins were associated to reactions that are in trade-off for some of the carbon sources. We found that the proteins that are associated with reactions in relative trade-offs are invariant to the change in the lower bound imposed on growth. The exception include: (i) malate dehydrogenase (E.C. 1.1.1.37) which, for lower bounds of 95% or 99% of the optimum growth, was found to be associated to some reactions in relative trade-off; however, it is not involved in any trade-offs at the lower bound of 90%, and (ii) L-lysine:tRNALys ligase (AMP-forming) (E.C. 6.1.1.6), that is associated to reactions in trade-off only at a lower bound of 90% of the optimal growth (Table S9). These findings indicate that different trade-offs become apparent as an organism approaches growth optimum. Lastly, like in *E. coli*, we found that reactions in trade-offs tend to be catalyzed by promiscuous enzymes (Table S5).

### 3.3 Relative flux trade-offs in *Arabidopsis thaliana* do not depend on key nutrients and the ratio of nitrate to ammonium

Next, we asked if the change in relative flux trade-offs with the environment can also be identified in metabolic models of other eukaryotes, like plants. It is known that the model plant *A. thaliana* can use different nitrogen sources (e.g. nitrate or ammonium) during autotrophic growth on the single carbon source CO_2_, and that the reference genotype Col-0 prefers nitrate over ammonium. To determine if the relative flux trade-offs depend on the different nitrogen sources, we employed the AraCore model, of *A. thaliana* metabolism, with three biomass reactions corresponding to optimal nitrogen (light-limiting), limiting nitrogen, and limiting carbon growth conditions [19]. In addition, for each of the three biomass reactions, we also considered three different nitrogen availability scenarios, namely only from nitrate, only from ammonium, or in equal proportion of nitrate and ammonium (50:50) [31]. We also investigated three sets of steady-state flux distributions, determined by a lower bound on growth given by 90%, 95%, and 99% of the optimum, determined by FBA [20]. Altogether, these variations in boundary conditions resulted in the investigation of relative trade-offs with respect to growth for 27 cases of the *A. thaliana* model.

Intriguingly, by applying FluTOr, we found that the set of 42 reactions participating in relative flux trade-offs was invariant with the change in the lower bound on growth between 90% and 99% of the optimum, irrespective of the biomass reaction used and of the nitrogen source used (Table S10). Altogether, 99% of all reactions were not blocked in all cases, and that 7.7% (i.e. 42) of reactions were in a relative trade-off (see Tables S10 and S11). This result indicate that the used model has a relatively small flexibility in achieving (sub)optimal growth, whereby the presence of only few alternative pathways leads to invariance of the identified relative trade-offs in the different test cases.

We found that altogether three and six of the 63 metabolic subsystem in the *A. thaliana* metabolic model were always and sometimes in trade-offs (see definitions in Section 3.2). Interestingly, the amino acid synthesis subsystems as well as the export subsystems were never in trade-off. This is in line with the expectation that a diversion of flux away from amino acids, as basic building blocks of biomass, or via export would lead to decreased growth. Three metabolic subsystems, including: cytidine triphosphate (CTP) synthesis, guanosine monophosphate (GMP) synthesis, and thioredoxins (THF) recycling, were always in trade-offs. However, some reactions in the subsystems related to nucleotide metabolism, tetrahydrofolic acid (TRX) recycling, inosine monophosphate (IMP) synthesis, and uridine monophosphate (UMP) synthesis, import, and transport were sometimes in trade-offs (Table S12 and Fig. S1). This result implies that an increase of the flux through any of the 42 reactions in trade-offs in the case of suboptimal growth would lead to increase in growth until the optimum (from FBA) is reached. At the optimum, the identified relative flux trade-offs either cannot be identified or become absolute trade-offs. Therefore, the concepts of absolute and relative trade-offs provide the means to better understand the space of alternative (sub)optimal flux distributions.

Comparison of the reactions in relative trade-offs with respect to growth based on the metabolic network of *A. thaliana* (Table S13) with published phenotypes on growth indicated that: (1) *guanylate kinase 3, chloroplastic* is required for optimal growth [32] and is also required for acclimation to nitrogen limitations [33], (2) overexpression of the genes encoding nucleoside diphosphate kinase leads to enhanced growth in poplar [34] and *alfalfa* [35], overexpression of the bifunctional *dihydrofolate reductase-thymidylate synthase 1* leads to delayed development, without differences in biomass and rosette size at the compared times with wild-type, suggesting larger final biomass [36]. These findings demonstrate that the predictions of the approach for Arabidopsis are partly in line with the existing experimental evidence.

Further, We found that 11% (i.e. 26) of the 225 proteins appearing in gene-protein-reaction rules were associated with reactions that were in trade-offs. In addition, only two proteins, *nucleoside diphosphate kinase* and *adenylosuccinate lyase* catalyzed reactions that were not in any trade-off (Table S13). Altogether, the findings from the analysis of the *A. thaliana* model suggested that the relative trade-offs reflect the specifics of the modeled reactions and pathways. In contrast to the scenarios with different carbon sources, that impose larger flux redistributions, the availability of ammonium or nitrate are restricted to specific pathways and, thus, result in universal trade-offs over all nitrogen availabilities scenarios considered.

### 3.4 Relations to relative trade-offs to partially and directionally coupled reactions

The proposed concept of relative flux trade-offs appears to be related to the concepts of directional and partial reaction couplings. Therefore, in the following we aimed to determine the extent to which relative trade-offs provide new insights in comparison to directionally and partially coupled reactions. From the formulation of FluTOr, it is clear that reactions in relative trade-off with respect to growth, must satisfy that $\sum_{i=1}^{n-1}\alpha_{i}v_{i}=v_{\mathrm{bio}}$. Therefore, non-zero flux through any of the reactions in a relative trade-off implies non-zero flux through the biomass reaction; moreover, non-zero flux through the biomass reaction may imply non-zero flux through some of the reactions in the relative trade-off. As a result, reactions in relative trade-offs are partially or directionally coupled to growth. However, the converse does not apply, i.e. not all reactions that are directionally or partially coupled to the biomass reaction need to be in a relative trade-off. This is the case since reactions that are in relative trade-offs with respect to growth need to satisfy additional conditions imposed in the formulation of FluTOr.

**Fig. 4:**
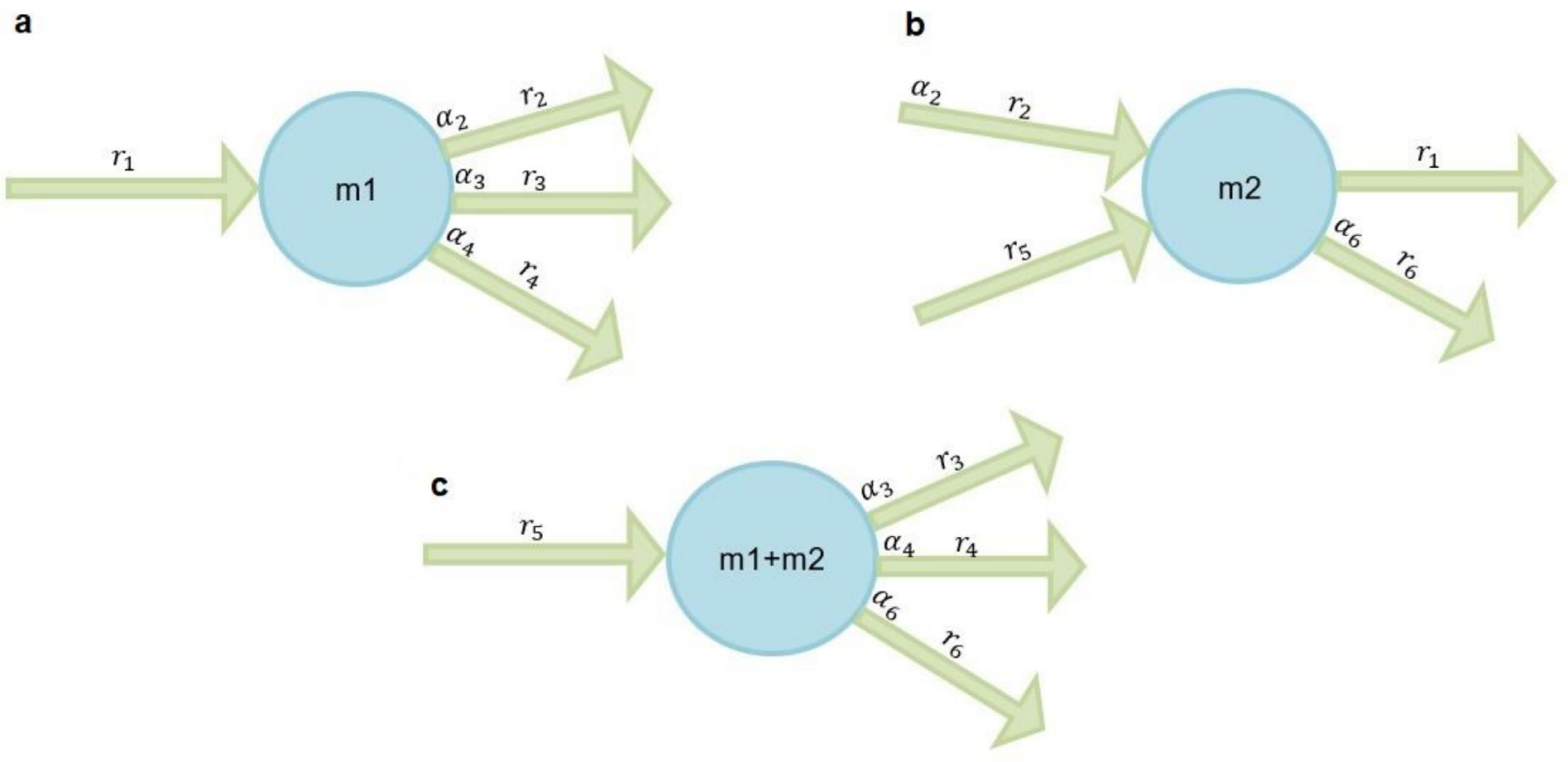
**Relative flux trade-offs and QFCA. (a) Directionally coupled equation.** The figure shows a relative trade-off between irreversible reactions ${\;r}_{2}\text{,}\;r_{3}$, and $r_{4\;}$ due to the directionally coupled equation ${\;v}_{1}={\alpha_{2}v}_{2}+\alpha_{3}\;v_{3}+\alpha_{4}\;v_{4}$*. (*b) Two reactions ${\;r}_{2}$ and ${\;r}_{5}$ produce metabolite $m_{2}$ and two reactions ${\;r}_{1}$ and ${\;r}_{6}$ consume it. (c) The summation of the stoichiometric matrix rows corresponding to ${\;m}_{1}$ and ${\;m}_{2}$ results in the equation ${\;\alpha_{3}v}_{3}+\;{\alpha_{4}v}_{4}+\;{\alpha_{6}v}_{6}=\;v_{5}$*,* yielding a relative trade-off*.* The latter is referred to as a merged directionally coupled equation.

To compare relative trade-offs with findings from QFCA, we relied on the notion of a directionally coupled equation (DCE). To illustrate a DCE, suppose there is a metabolite $m_{1}$ with a reaction $r_{1}$ producing it, and three reactions, $r_{2},r_{3}$, and $r_{4}$, consuming this metabolite with molarity $\alpha_{2},\alpha_{3}$, and $\alpha_{4}$, respectively (Fig. 4a). If all four reactions are of variable flux, then at steady state it must hold that $v_{1}={\alpha_{2}v}_{2}+\alpha_{3}v_{3}+\alpha_{4}v_{4}$, which represented a relative trade-off. This relation forms a directionally coupled equation (DCE), since non-zero fluxes through any of the reactions $r_{2}\text{,}\;r_{3}$, and $r_{4}$ implies non-zero flux through reaction $r_{1}$, rendering all three reactions directionally coupled to $r_{1}$. In contrast to QFCA, to adequately model relative trade-offs, here we allowed only for irreversible reactions (carrying non-zero flux) to appear in a DCE. In addition, we note that this equation can be extended by finding more relative trade-offs by merging other metabolites. For instance, if there is a second metabolite $m_{2}$ with two incoming irreversible reactions $r_{2}$ and $r_{5}$ and two outgoing irreversible reactions $r_{1}$ and $r_{6}$ , then the equation ${\alpha_{2}v}_{2}+v_{5}={\alpha_{1}v}_{1}+\alpha_{6}v_{6}$ holding at steady-state (Fig. 4b) . By summation of the two equation we a new relative trade-off ${\alpha_{3}v}_{3}+{\alpha_{4}v}_{4}+{\alpha_{6}v}_{6}=v_{5}$ (Fig. 4c), leading to a merged DCE.

**Fig. 5:**
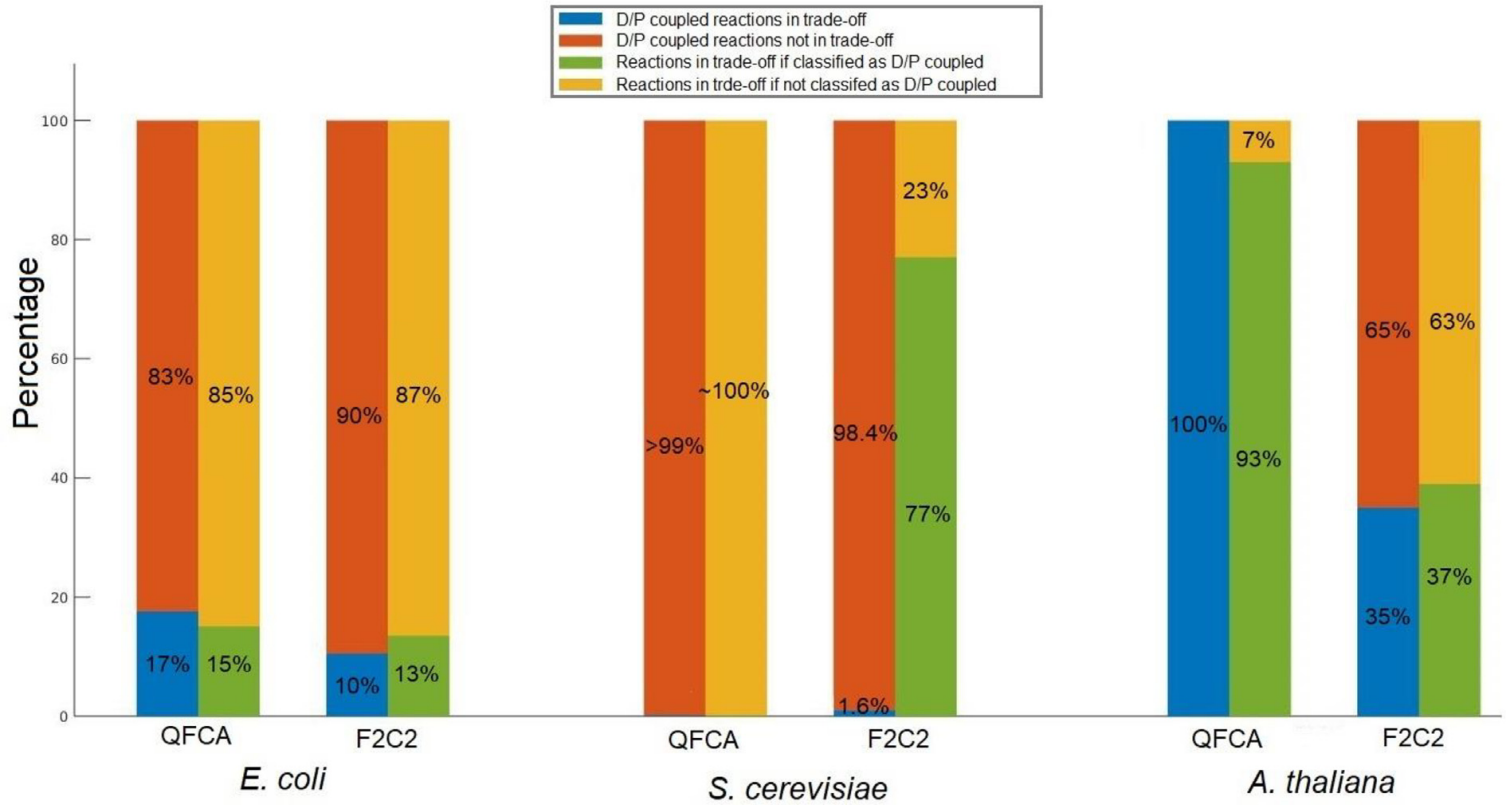
**Partially or directionally coupled reactions in trade-off in the models**. Shown is the percentage of reaction that are in relative trade-off and whether or not they are partially (P) or direction (D) coupled to the biomass reaction. It also shows the percentage of P/D reactions to the biomass reaction that (do not) participate in relative trade-offs.

To determine the degree to which the findings from FluTOr coincide with those obtained from FCA, we compared the findings with those from QFCA and F2C2 under the same constraint on growth imposed in FluTOr. As illustrated on Fig. 5, for the model of *E. coli* only 17% and 10% of directionally or partially coupled reactions to biomass were in the identified relative trade-offs, as determined by QFCA and F2C2, respectively. Further, the reactions in relative trade-offs represented only 15% and 13% of partially or directionally coupled reactions. For the model of *S. cerevisiae*, on average only 0.18% and 1% of partially/directionally coupled reactions, determined by QFCA and F2C2, respectively, took part in relative trade-offs. As a result, FluTOr resulted in more partially or directionally coupled reactions in comparison to QFCA and F2C2 with the same imposed constraints. Further, among the reactions that were in relative trade-off, on average 1.6% and 77% were partially or directionally coupled detected per QFCA and F2C2 method (Fig. 5).

Applying QFCA with the same constraints in the case of *A. thaliana* models identified 6.4% of reactions to be partially or directionally coupled to the biomass reactions, and all of them were identified by FluTOr. In addition, by applying FluTOr we found 7% more partially or directionally coupled reactions to biomass. The results based on the F2C2 method differed substantially; over the considered test cases, on average, 7.2% of reaction were found to be partially or directionally coupled to the biomass reactions. FluTOr found only 35% of them in the identified relative trade-offs. In addition, FluTOr also identified 61% partially or directionally coupled reactions to the biomass reaction that were not identified by F2C2 (Table S14, Fig. 5). Altogether, our findings demonstrate that the concept of relative trade-offs shows subtle difference to partially and directionally coupled reactions, and adds to better understanding of operational constraints on metabolic functionalities.

## 4 Conclusion

While there is growing evidence that trade-offs affect the phenotypes attainable through evolution, there is little understanding of the biochemical constraints that contribute to the emergence of molecular trade-offs. Since biochemical components do not exist in isolation, but are integrated in various cellular networks, it is expected that aspects of the network structure, given by the set of all interactions in which the components take place, along with principles of network operation can determine the presence of particular trade-offs.

Large-scale metabolic models gather the entirety of known metabolic reactions and their function can be systematically analyzed and by approaches from the constraint-based modeling framework. Here, we expand this framework by proposing FluTOr, a constraint-based modeling approach that identifies relative trade-offs between fluxes with respect to a flux of a specified reaction. FluTOr is more versatile than FluTO, the approach for identification of absolute flux trade-offs, since it does not express a non-negative linear combination of fluxes in terms of a flux that is invariant with respect to the environment. Further, since relative trade-offs are given by non-negative linear combinations of fluxes that amount to the flux of the specified reaction, FluTOr also provide (upon mild assumptions) the means to specify overexpression targets aimed at optimization of the objective. As a result, the identified relative trade-offs provide insights in the relation between constraints that shape trade-offs and optimization of cellular tasks. For the case of *A. thaliana* our prediction for overexpression based on the identified relative trade-offs are mostly in line with the limited evidence available on overexpression lines.

By applying the proposed approach to three large-scale metabolic models in simulations of different environments, we demonstrated that relative trade-offs with respect to growth (modeled as the flux through the biomass reaction) are often condition-dependent, having important implications for optimization of fitness. Our findings corroborate that the coordinated activation of central metabolic processes underlie the relative flux trade-offs that we also found are species-specific. Future applications of FluTOr will examine the extent of interlinking between relative trade-offs due to optimization of multiple cellular tasks as well as connections to resource allocation in the context of protein-constrained metabolic models.

## CRediT authorship contribution statement

**Seirana Hashemi:** Data curation, Formal analysis, Investigation, Methodology, Software, Validation, Visualization. **Zahra Razaghi-Moghadam:** Data curation, Investigation, Methodology, Validation, Writing – review & editing. **Roosa A.E. Laitinen:** Funding acquisition, Supervision, Writing – review & editing. **Zoran Nikoloski:** Conceptualization, Formal analysis, Funding acquisition, Investigation, Methodology, Supervision, Validation, Visualization, Writing – original draft, Writing – review & editing.

## Declaration of Competing Interest

The authors declare that they have no known competing financial interests or personal relationships that could have appeared to influence the work reported in this paper.
