# Supplementary material for: Relative flux trade-offs and optimization of metabolic network functionalities
Source: Comput Struct Biotechnol J. 2022 Jul 26;20:3963–71. doi: 10.1016/j.csbj.2022.07.038 (PMC9340536; doi:10.1016/j.csbj.2022.07.038)

**Supplementary Note. The problem formulation.**

We select a reaction from $R$,and call it $v_{j}$. Then we are looking for at least two reactions from $R-\{r_{j}\}$; $b_{j}v_{j}+\sum b_{i}v_{i}=0, r_{i}\in R-\left\{ r_{j} \right\},0<b_{j}\leq M \&-M\leq b_{i}\leq0$.

The MILP formulation excludes all previously found trade-offs, which is achieved by using integer cuts. The blocked reactions have been removed from the model. Reaction are non-blocked irreversible. The problem solves in this way.

$$min\sum-b_{i}{,ifv}_{i}\in R-\{r_{j}\}$$

s.t.

$$\mathbf{N}_{\boldsymbol{m\times r}}\mathbf{v}_{\boldsymbol{r\times1}}=\boldsymbol{0}$$

$$\mathbf{k}_{\boldsymbol{1\times m}}\mathbf{N}_{\boldsymbol{m\times r}}\mathbf{=}\mathbf{b}_{\boldsymbol{1\times r}}$$

$$0<b_{j}\leq M,for v_{j}$$

$$-M\leq b_{i}\leq0,ifv_{i}\in R-\{r_{j}\}$$

$${0\leq b}_{i}+Ms_{i}\leq M-1,ifv_{i}\in R-\{r_{j}\}$$

$$s_{i}\in\left\{ 0,1 \right\}, for v_{i}\in R-\{r_{j}\}$$

$$M=100$$

**Figure S1: Subsystems connectivity based on pair of reactions in trade-off in A. thaliana.** Nodes are the subsystems and weight of the edges indicate the number of pairs of reactions in relative trade-offs belong to the two connected subsystems.


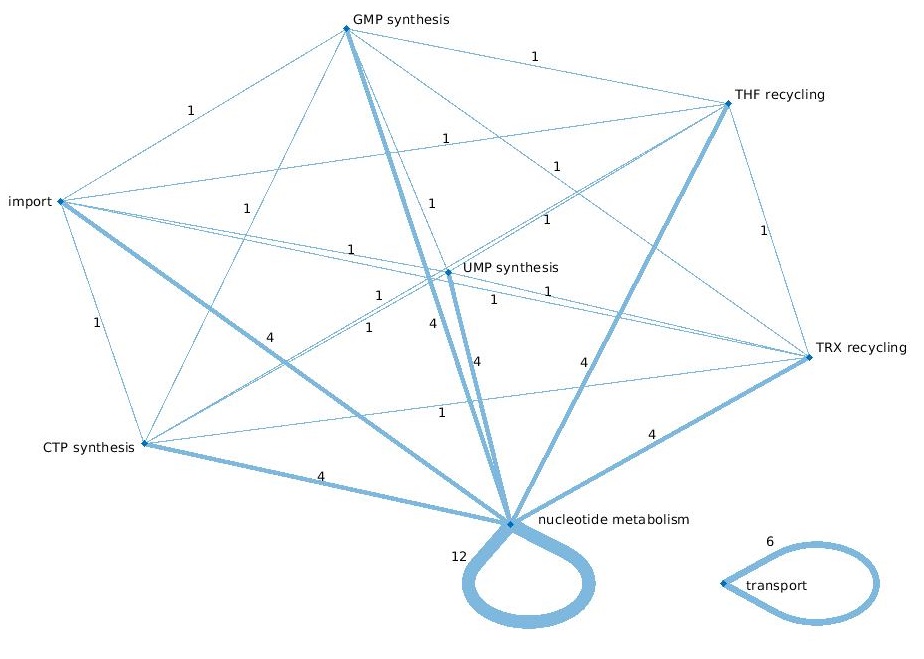

Supplement: Supplementary data 1 [file mmc1.docx]
